# Dendritic Nanotheranostic for the Delivery of Infliximab: A Potential Carrier in Rheumatoid Arthritis Therapy

**DOI:** 10.3390/ijms21239101

**Published:** 2020-11-30

**Authors:** Tamara Rodríguez-Prieto, Borja Hernández-Breijo, Miguel A. Ortega, Rafael Gómez, Javier Sánchez-Nieves, Luis G. Guijarro

**Affiliations:** 1 Department of Organic and Inorganic Chemistry, Chemical Research Institute “Andrés M. Del Río” (IQAR), University of Alcalá, 28805 Madrid, Spain; tamara.rodriguezp@edu.uah.es (T.R.-P.); rafael.gomez@uah.es (R.G.); javier.sancheznieves@uah.es (J.S.-N.); 2 Ramón y Cajal Health Research Institute (IRYCIS), 28034 Madrid, Spain; luis.gonzalez@uah.es; 3 Networking Research Center on Bioengineering, Biomaterials and Nanomedicine (CIBER-BBN), 28029 Madrid, Spain; 4 Immuno-Rheumatology Research Group, IdiPaz Hospital Universitario La Paz, 28046 Madrid, Spain; borja.hernandez@idipaz.es; 5 Department of Medicine and Medical Specialties, Faculty of Medicine and Health Sciences, University of Alcalá, Alcalá de Henares, 28801 Madrid, Spain; 6 University Center for the Defense of Madrid (CUD-ACD), 28047 Madrid, Spain; 7 Tumour Registry, Pathological Anatomy Service, University Hospital Príncipe de Asturias, 28805 Alcalá de Henares, Spain; 8 Department of Systems Biology, Faculty of Medicine and Health Sciences (Networking Research Center on for Liver and Digestive Diseases (CIBEREHD)), University of Alcalá, 28801 Alcalá de Henares, Spain

**Keywords:** dendrimer, antibody, theranostic, carrier, rheumatoid arthritis

## Abstract

Antibodies are macromolecules that specifically recognize their target, making them good candidates to be employed in various therapies. The possibility of attaching a drug to an immunoglobulin makes it possible to release it specifically into the affected tissue as long as it overexpresses the target. However, chemical coupling could affect the functionality (specificity and affinity) of the antibody. It has been observed that the use of intermediaries, such as dendrimers, could resolve this issue. Because carbosilane dendrimers have aroused great interest in the field of biomedicine, this report describes the synthesis of an anionic carbosilane dendrimer with a fluorochrome on its surface that then forms a conjugate with an antibody. It has been used as immunoglobulin and infliximab, whose target is TNF-α, which is a cytokine that is overexpressed in the inflamed area or even in the blood of patients with autoimmune diseases, such as rheumatoid arthritis. In addition, the integrity and functionality of the antibody has been studied to see if they have been affected after the chemical coupling process.

## 1. Introduction

Rheumatoid arthritis (RA) is an incapacitant autoimmune disease (AID) characterized by bone and cartilage damage, joint inflammation, pain and stiffness, with a prevalence estimated at about 1% worldwide [1,2]. Female gender, familiar history, genetic factors (shared epitope), and exposure to tobacco smoke are considered the major risk factors of RA. Additional current research also focuses on the role of mucosal inflammation and the microbiota [3]. Patients with RA frequently present further comorbidities, including cardiovascular diseases, infections, or osteoporosis, because of the advanced age of the individuals or the treatment received [4,5], importantly decreasing the quality of life of the affected person. Hence, it is necessary to research effective specific treatments directed not only to RA but also to ameliorating the risk of comorbidities in these patients. RA, along with other AIDs exhibiting an inflammatory component, such as ankylosing spondolitis or inflammatory bowel disease, are frequently treated with the antibody infliximab (IFX). IFX is a chimeric monoclonal Ab against TNF-α (tumor necrosis factor). TNF-α is a glycotoxic cytokine with important functions, such as promoting endothelium permeability, causing an increase in IgG, activating the supplement, promoting the migration of monocytes and T and B lymphocytes into the affected tissue, and enhancing the immune response. Therefore, a massive response of TNF can cause edema and the collapse of blood vessels and can participate in multiorgan failure [6]. Thus, in diseases where an uncontrolled increase in this cytokine occurs, treatment with anti-TNF-α has proven its efficacy.

Antibodies (Ab) or immunoglobulins are plasmatic proteins formed by two light and two heavy chains, presenting a carbohydrate component bound to aminoacidic residues. Abs are able to specific recognize almost all type of organic compounds [7,8]. As a natural system, Abs defend us from external dangers. However, their ability to identify different targets, have promoted its use in a broad spectrum of pathologies, mainly in the fields of oncology, rheumatology, and inflammatory diseases [9]. 

The production and discovery of new Abs in clinical therapy has presented an exponential growth in the recent years, currently valued at around 125 billons $ worldwide, and it is hoped that this will keep rising [10,11]. Ab may act directly through its binding to the specific target, inhibiting its function, or combined with additional conjugated drugs, hence addressing the proper objective—increasing the efficacy of the therapy [12,13]. Equally, the use of Ab has also reported its utility as a method of diagnosis and combined diagnosis and therapy or theragnosis [14,15]. On the other hand, a high cost-effectiveness ratio, cytotoxicity, and the lack of knowledge about the adequate dose are the major limitations of the use of Ab in therapy [16,17].

In this sense, dendrimers are nanosized synthetic macromolecules with a globular conformation and a monodisperse and hyperbranched structure. This well-defined scaffold is obtained through simple reactions that are repeated until the desired size is reached. Dendrimer synthesis can be carried out using two distinct methods, convergent and divergent methods. The convergent method is based on synthesizing the dendrimer from the outside to the core, while in the divergent method the dendrimer is synthesized from the nucleus outwards [18]. 

Generally, three parts can be differentiated in its structure: the core or focal point, where the branches emerge; the intermediate zone, which determines the generation of the dendrimer; and the multivalent surface, where the functional groups are located. Because they can have a variable number of branches, one way to classify dendrimers, among others, is depending on the generation they present [19].

Dendrimers have been synthesized with different skeletons, which will clearly influence their physicochemical properties. Among the most common scaffolds are poly (amido amine) dendrimers (PAMAM), polypropylene dendrimers (PPI), polyesters, triazines, and ones with phosphorus and silicon atoms in their structure [19]. In addition to the internal structure, the peripheral functional groups also determine the application to which they will be intended—for example, dendrimers with cationic groups are used as antibacterial agents [20], while those with anionic groups are used as antivirals [21]. At the same time, the multivalence they present on the periphery allows in the same dendrimer to possess different functional groups, with the possibility of coupling a wide variety of molecules such as drugs, fluorochromes, nucleic acids, etc. Due to their versatility, multivalence. and structural precision, dendrimers have aroused a great interest in their application in biomedical sciences. However, in order to be used in biomedicine they must meet a number of requirements. In this sense, it should be noted that non-toxic, cross biological barriers are stable and remain in circulation for as long as necessary to have clinical effect, in addition to being able to interact specifically with the pharmacological target. Thus far, dendrimers have been used, among others, as drug and nucleic acid transporters, as therapeutic agents or as contrast agents, as well as in nanodiagnosis [22].

Carbosilane dendrimers are dendrimers with a framework made of C-C and C-Si bonds. These types of bonds are of a very low polarity and, hence, the carbosilane framework is hydrophobic, in contrast with other dendrimers such as PAMAM, PPI, or polyesters. This hydrophobicity enables the interaction with cell membranes and carbosilane dendrimers have been employed in a variety of biomedical applications [23].

Due to the great development of the use of dendrimers in biomedicine, this work focuses on obtaining an anionic carbosilane dendrimer for subsequent binding to a therapeutic antibody in order to obtain a conjugate of possible clinical application. Several studies have focused on this goal, as antibodies are molecules that specifically interact with their target, and this is interesting when directing a drug towards the affected tissue. It would be important to be able to attach to such specific molecules a drug or any other drug with a therapeutic function (e.g., anticancer, anti-inflammatory, etc.). It has been seen that the direct binding of a drug with an antibody can cause the antibody to become no longer functional. Therefore, the intervention of a dendrimer, such as a mediator. between the antibody and the medicine, would be interesting if the functionality of the antibody was not altered. It has also been seen that dendrimers may have a radioactive isotope attached [24], so it is foreseeable that the conjugation of this type of dendrimers with antibodies could be used in chemotherapy. This type of studies have been carried out on PAMAM dendrimers, which as previously mentioned were the first to be synthesized and have therefore been the most studied. Currently such dendrimers are available in the trade due to their high solubility and biocompatibility, which makes them good candidates for transporting ligands, fluorochromes, and drugs, among other molecules [25].

This work focuses on the use of carbosilane dendrimers, which have a skeleton formed by Si-C bonds, so they are stable, lipophilic, and inert. This group of dendrimers are very versatile and are being developed in the field of biomedicine for use as drugs, nucleic acids, and fluorochromes transporters. In this work, a third generation anionic carbosilane dendrimer is used with the aim of forming a conjugate with an antibody.

In this work, infliximab has been used as a model to develop a protocol to conjugate an antibody to a polyanionic carbosilane dendrimer.

## 2. Results

### 2.1. Synthesis and Characterization of FITC-Labelled Dendrimer

**Figure 1:**
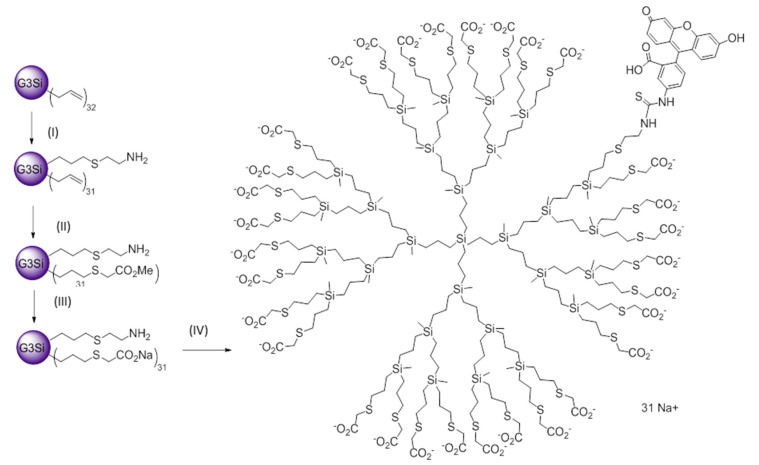
Synthetic route towards FITC-labelled dendrimer IV. (I) HS(CH_2_)_2_NH_2_·HCl, MeOH/THF, DMPA, UV, 30 min; (II) HS(CH_2_)_2_CO_2_Me, MeOH/THF, DMPA, in situ, UV, 6 h; (III) NaOH/MeOH, 2 h; (IV) FITC, TEA, DMF, 12 h.

The anionic carbosilane dendrimer labelled with fluorescein isocyanate (FITC) was synthesized from the precursor dendrimer G_3_SiAl_32_ (I), bearing 32 allyl groups in the periphery, through a 3-step route (Figure 1). In the first step, dendrimer I was reacted with cysteamine hydrochloride through a UV-initiated thiol-ene reaction in MeOH/THF, initiated through the photoinitiator DMPA. A 1:1 stoichiometry was employed, aiming to substitute a single peripheral group. The reaction is completed in 30 min, leading to dendrimer I (non-isolated). A second thiol-ene reaction was performed in situ, after the addition of methyl thioglicolate in a 1:31 stoichiometry and employing the same reaction conditions. After 6 h irradiation, dendrimer G_3_Si(SC_2_H_4_NH_2_)(SCH_2_COOCH_3_)_31_ (II) was obtained and isolated as a yellow oil. In a third step, dendrimer II was reacted with NaOH (1:31) in MeOH, at room temperature for 2 h. The ester groups were thus converted into anionic carboxylate moieties, leading to the white solid G_3_Si(SC_2_H_4_NH_2_)(SCH_2_COO^−^Na^+^)_31_ (III) after nanofiltration (Ultracel 3 kDa Ultrafiltration Discs, San Luis, AZ, USA), until a neutral pH was obtained in dissolution. In the final step, FITC was reacted with dendrimer III in DMF, for 12 h at r.t. and protected from light. The reaction was monitored through Kaiser Test [26], a colorimetric assay which confirms the presence or absence of primary amines. Once the –NH_2_ was completely reacted, the solvent was evaporated and the solid was washed with ethanol. The resultant dendrimer (G_3_Si(SC_2_H_4_NHFITC)(SCH_2_COO^−^Na^+^)_31_ (IV) was isolated as an orange solid.

The complete synthetic route to dendrimer IV was monitored through a range of different techniques, including Nuclear Magnetic Resonance (^1^H-NMR), Fourier-Transformed Infrared Spectroscopy (FTIR), Ultraviolet-Visible Spectrophotometry (UV-Vis). 

**Figure 2:**
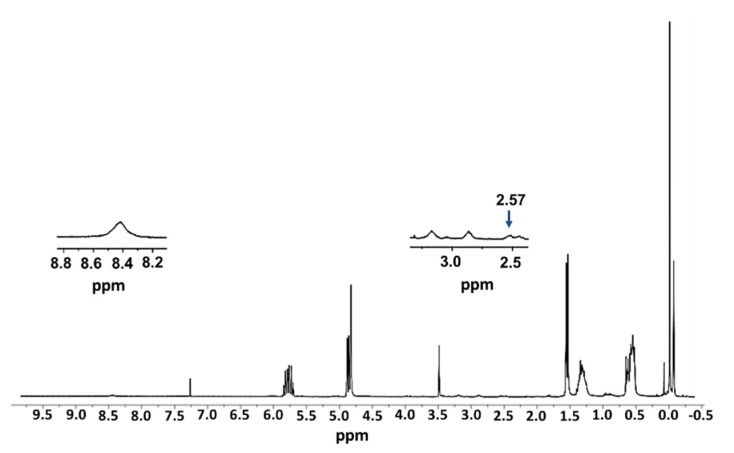
^1^H-NMR of G_3_Si(Allyl)_31_(SC_2_H_4_NH_3_Cl) (I) in CDCl_3_.

In the ^1^H-NMR of compound I (Figure 2, enlargement), the signals corresponding to the cysteamine fragment, 8.4 ppm (3 H, s, NH_2_), 3.22 ppm (2 H, t, C*H*_2_NH_2_), 2.92 ppm (2 H, t, SC*H*_2_CH_2_NH_2_), and the rest correspond to the internal skeleton of the dendrimer. However, it is worth noting the appearance of a new signal at 2.57 ppm (2 H, t, SiCH_2_CH_2_C*H*_2_S), which allows us to know that the reaction has ended. In Figure 2 the signs of cysteamine are difficult to appreciate as it should be noted that a single allyl group is reacting, and therefore the peaks are less intense.

**Figure 3:**
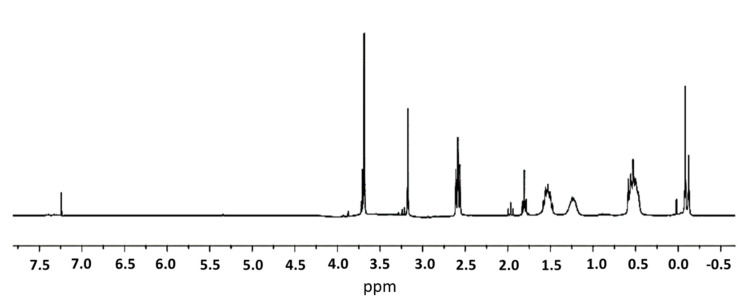
^1^H-NMR of G_3_Si(SC_2_H_4_NH_2_)(SCH_2_CO_2_CH_3_)_31_ (II) in CDCl_3_.

In the ^1^H-NMR of compound II (Figure 3), it is important to highlight the disappearance of signals corresponding to the allyl groups, which appeared around 4.8 and 5.8 ppm, indicating that the reaction had ended. In addition, we can observe the signals of the dendritic skeleton and the corresponding new signals of methyl glycollate 3.72 ppm (93 H, s, OC*H*_3_) and 3.20 ppm (62 H, s, SC*H*_2_CO).

**Figure 4:**
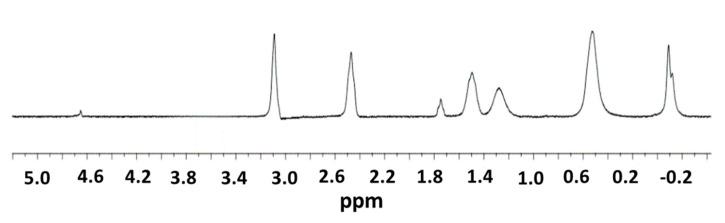
^1^H-NMR of G_3_Si(SC_2_H_4_NH)(SCH_2_CO_2_Na)_31_ (III) in D_2_O.

The ^1^H-NMR of compound III (Figure 4) differs from the spectrum of compound II only in the signal at 3.72 ppm (93 H, s, OCH_3_). This signal disappears when you react with NaOH because the methyl groups of the ester are removed. That is why, in the ^1^H-NMR spectrum of compound III, we no longer observe that signal at 3.72 ppm, so we can say that the reaction is over.

**Figure 5:**
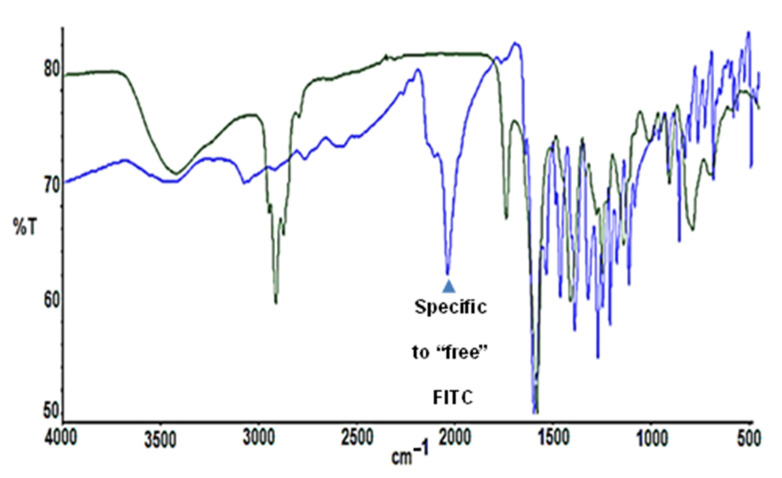
IR spectrum of FITC (blue) and compound IV (green).

In the case of compound IV, it is very difficult to follow the reaction using the ^1^H-NMR spectrum, since it should be noted that we are only modifying one branch of thirty-two that has the entire dendrimer. In addition, the new signals corresponding to the reagents that have been incorporated in the successive steps of synthesis overlap with those that would determine the binding of FITC to compound III. That is why IR, VIS-UV, and Kaiser Test spectroscopy were used. Figure 5 shows the IR spectrum of FITC (blue) and compound IV (green), where it should be noted that the main signal that allows the coupling of compound III to be identified to the FITC, is the disappearance of the peak at 2180 cm^−1^. This signal corresponds to the asymmetric voltage of the S-C-N group that the FITC possesses before joining it with compound III, so that once the reaction is complete this signal disappears (Figure 5, green).

**Figure 6:**
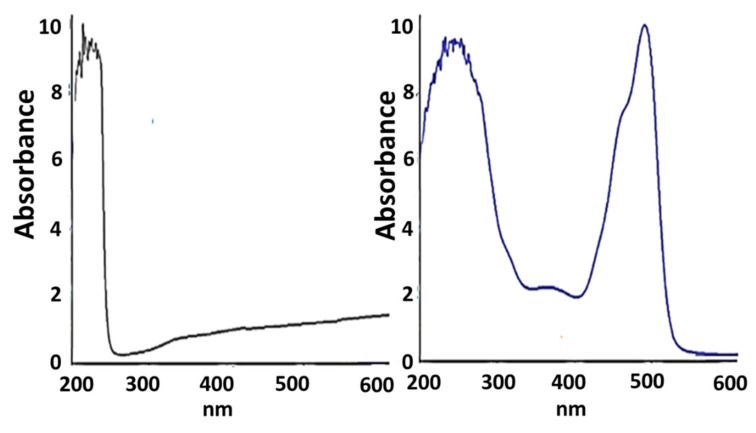
UV/VIS spectrum of compound III (**left**) and compound IV (**right**).

**Figure 7:**
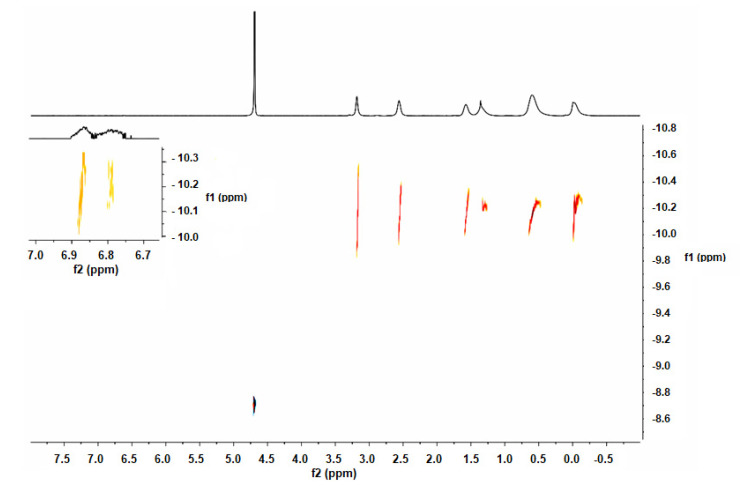
DOSY-^1^H-NMR of G_3_Si(SC_2_H_4_NH FITC)(SCH_2_CO_2_Na)_31_ (IV) in D_2_O.

As for the VIS-UV spectrum, we can determine the presence of FITC in the resulting compound IV, as a peak appears around 500 nm corresponding to the conjugated ring system of the same. In Figure 6 (left) we see the VIS-UV spectrum of compound III, whose peaks at approximately 230 nm correspond to the skeleton of the dendrimer, due to the heteroatoms it possesses. In Figure 6 (right), we see the spectrum of compound IV, where, in addition to the peaks that previously appeared, the one corresponding to the FITC molecule at 494 nm also appears. We performed two-dimensional diffusion-ordered spectroscopy, DOSY-^1^H-NMR, to confirm the molecular structure of compound IV (Figure 7). The presence of new signals (Figure 7, enlargement) confirms the presence of FITC.

### 2.2. Molecular Characterization of Infliximab and Compound IV Using SDS-PAGE

**Figure 8:**
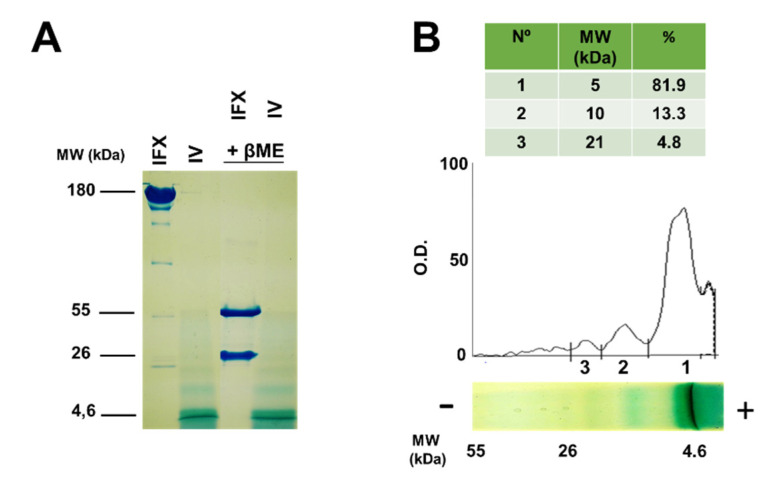
(**A**) Electrophoresis of infliximab (IFX) and compound IV using SDS-PAGE. (**B**) Densitometric analysis of electrophoresis of compound IV in absence of βME (β-mercaptoethanol). O.D. = optical density.

In order to further confirm the purity and molecular weight of infliximab and compound IV, SDS-PAGE was employed under non-reducing and reducing (in the presence of β-mercaptoethanol) conditions, and subsequently stained with Coomassie Blue. The advantage of using this technique is that SDS will solubilize the dendrimer while coating it with negative loads, in this way it will migrate in the polyacrylamide network depending on its molecular weight. Figure 8A shows the following bands 160 kDa and 5 kDa corresponding to infliximab and to dendrimer respectively in non-reducing conditions. In reducing conditions, we observed two bands of 55 and 26 kDa, corresponding to infliximab, whereas compound IV did not change in these conditions. The electrophoresis of compound IV revealed other minor bands that appear at higher molecular weights. Densitometry of the bands (Figure 8B) confirmed the percentage of the aggregates, with 13.3% dimers and 4.8% trimers. The possibility of obtaining aggregates in synthesis is low because there is only one amine group per thirty-one carboxilate groups.

### 2.3. Conjugation of Infliximab to Labelled Dendrimer

**Figure 9:**
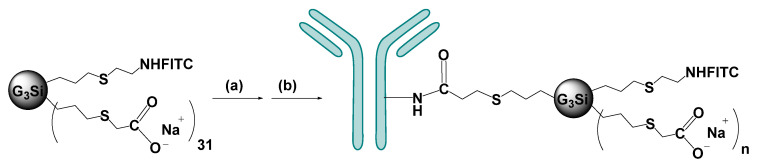
Conjugation between compound IV and infliximab. Formation of dendriplex IFX-IV (a) NHS, EDCI, MES (100 mM), 1 h; (b) infliximab, HEPES (100 mM), 24 h.

The antibody IFX was covalently bound to the dendritic compound IV through an amide bond. (Figure 9). First, the carboxylate groups were activated with 2-(N-morpholine) ethanol sulfonic acid (MES) 100 mM (pH 4.8) for 15 min, protected from light. Then *N*-hydroxysuccinimide (NHS) and 1-ethyl-3-(3-dimethylaminopropyl) carbodiimide (EDCI) were added to the solution in a 1:16 and 1:8 stoichiometry, respectively. We followed the procedure for the activation of carboxylates using EDCI/NHS indicated by the manufacturer (Thermo scientific, Waltham, MA, USA). After 1 h reaction in dark, compound IV was isolated through centrifugation and reacted with IFX in HEPES buffer 100 mM at different proportions (ratio IV:IFX = 1:1; 2:1; 5:1 and 10:1).

**Figure 10:**
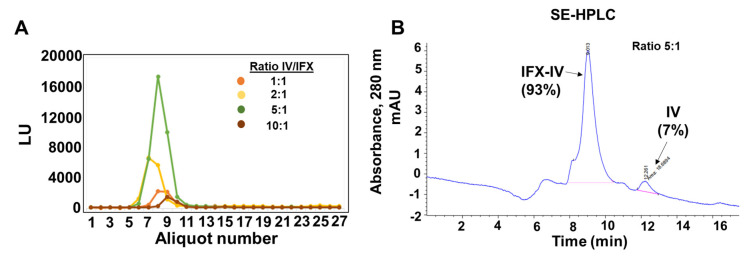
(**A**) Sephadex G25 chromatographic profiles of IFX-IV complexes. (**B**) SE-HPLC chromatographic profile of IFX-IV complex obtained at the ratio 5:1.

The IV-IFX conjugates were purified through dialysis (Slide-A-Lyzer Dialysis cassette 10,000 MWCO, Thermo Scientific, Waltham, MA, USA) for 24 h and Sephadex G25 chromatography (Thermo Fisher) in PBS. In the Figure 10A, we showed the chromatographic profiles of the IFX-IV complexes obtained at the different ratios studied. The fluorescence of the complexes present in the fractions was measured at λ = 494 nm (Figure 10A). A single peak appears in all conditions tested that correspond to the complex IFX-IV which correspond to the column exclusion volume (Vo). These data indicated, that small molecules (IV and FITC) that were in excess have been eliminated through the dialysis process. Subsequent experiments were conducted with the complex obtained at the ratio 5:1, because we obtained the best fluorophore brightness index score. In the same coupling conditions (ratio 5:1), the IFX-IV complex represented around 97% whereas compound IV represented 3% (Figure 10B). This set of experiments were used to obtain the protein concentration of IFX-IV complex, using the theoretical extinction coefficient for human immunoglobulin and the integrated peak areas obtained by the Agilent ChemStation software according to the equation:

(1) $$c\;=\;\frac{\left(A\right)}{\left(d\;\times \;1000\;\times \;\varepsilon \right)}$$

where c = concentration (mg/mL), A = area (mAU × mL) × peak volume for IFX-IV, d = optical path (cm), and ε = theoretical extinction coefficient (mg^−1^ cm^−1^). ε = 13.6 mg^−1^ cm^−1^ for human immunoglobulin. Using these parameters, we obtained for the IFX-IV complex a value of protein concentration of 18.10 ± 1.8 µg/mL.

**Figure 11:**
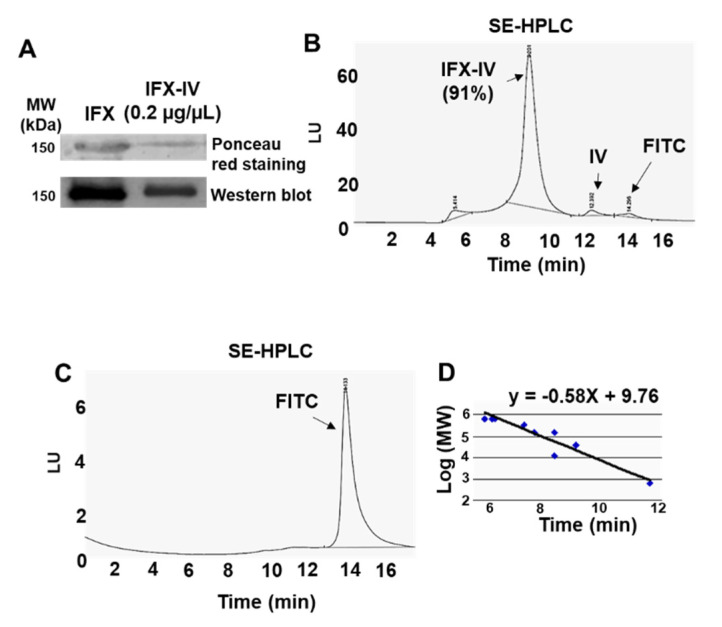
(**A**) SDS-PAGE electrophoresis and immunoblot of IFX-IV complex. (**B**) SE-HPLC chromatogram of IFX-IV conjugate. (**C**) HPLC chromatogram of FITC. (**D**) SE-HPLC calibration curve.

**Figure 12:**
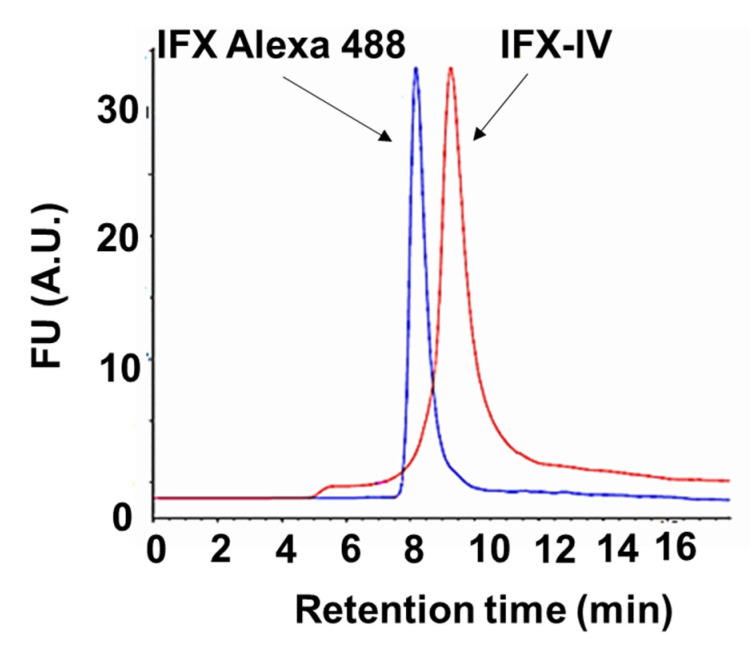
SE-HPLC chromatogram of compound IV-IFX conjugate (red) and IFX Alexa488 conjugate B (blue).

The purity and molecular weight of compound IV-IFX were further confirmed through SDS-PAGE electrophoresis, immunoblotting, and SE-HPLC chromatography. We observed by electrophoresis under non-reducing conditions and subsequent Ponceau red staining a band at 150 kDa which is compatible with a monomeric IgG. The molecular weight was confirmed by immunoblot (Figure 11A). We confirmed a high purity (over >90%) of the IFX-IV complex using SE-HPLC (Figure 11B) and subsequent analysis by fluorescence detection techniques (λ = 494 nm). Minority peaks corresponding to IgG aggregates (retention time = 5.4 min), compound IV (retention time = 12.4 min) and FITC (retention time = 12.3 min) were occasionally detected (Figure 11C). The IFX-IV complex displayed a retention time of 8.9 ± 0.4 min which correspond to a range of 130–30 kDa after the interpolation in the calibration curve shown in Figure 11D. In previous data obtained in our laboratory [26] we observed for IFX-Alexa 488 a narrow peak in SE-HPLC and a retention time of 8.1 min compatible with a MW of 150 kDa. These data have been confirmed in present results and in Figure 12 we showed the comparison between IFX-IV complex (retention time = 8.9 min) and IFX-Alexa 488 (retention time = 8.1). These results suggest that the incorporation of compound IV increased the molecular heterogeneity of the complex and changed its hydrodynamic properties. In addition, the number of dendrimers coupled to IFX was determined. An average of 1.5 ± 0.4 dendrimers per IFX molecule was coupled.

### 2.4. Evaluation of Functionality of Compound IV-IFX through ELISA

**Figure 13:**
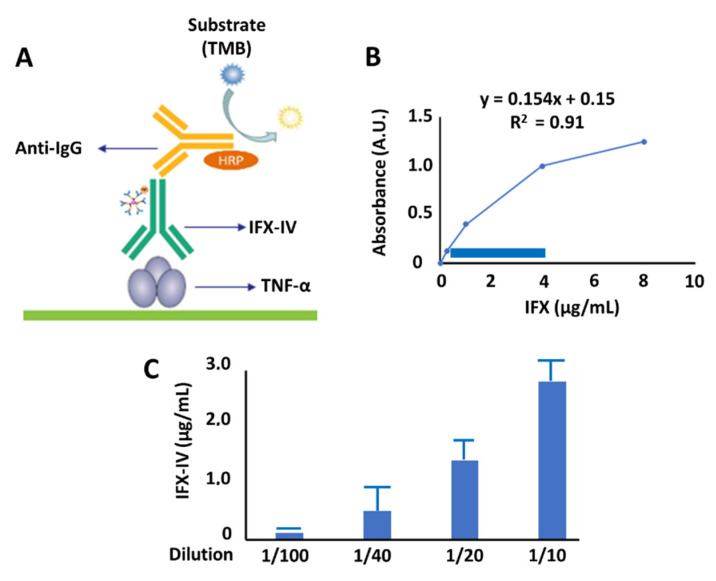
(**A**) ELISA experiment using TNF-α as a capture antigen. (**B**) Standard curve using IFX. (**C**) Compound IV-IFX concentration in a serial dilution.

Once it was assessed by SDS-PAGE and immunoblotting, the molecular integrity of IFX-IV was not significantly modified in the coupling process, we evaluated the affinity of IFX-IV complex against its target. For this purpose, we performed an ELISA experiment using TNF-α as capture antigen (Figure 13A). The objective of this set of experiments was to know whether the Fab region of IFX-IV complex had been modified or whether it remained able to recognize TNF-α. A calibration curve was carried out with known concentrations of infliximab (Figure 13B) The blue rectangle corresponds to the linear region of the standard curve. The diluted concentrations of IFX-IV complex were measured in the standard curve and are shown in Figure 13C. The concentration of IFX-IV obtained in the conjugation process described above was determined and corresponded to 13.06 ± 1.6 µg/mL). To see how it is possible that a considerable amount of compound IV is conjugated to the Fab domain, and in this way changes the biological functionality of the molecule, we have studied the ratio between the total protein concentration obtained by SE-HPLC and subsequent UV analysis at 280 nm (18.10 ± 1.8 µg/mL) and the concentration of functional IFX-IV obtained by ELISA method (13.06 ± 1.6 µg/mL). The ratio obtained corresponded to 72.1%.

In conclusion, the obtained IFX-IV complex recognizes the antigen (TNF-α) adsorbed to the ELISA plate and therefore maintains its functionality in a high proportion.

## 3. Discussion

Our study has described the procedures and steps to develop a conjugated carbosilane dendrimer with IFX. To our knowledge, there are still neither studies describing IFX-dendrimer conjugates nor clinical trials with this condition. Notwithstanding this, Pabari et al. [27] recently elaborated polyutherane-based nanoparticles to act as specific nanocarriers of IFX in some in vitro models. Their results showed a promising role of IFX-nanocarriers conjugates at a basic level, although further research is needed to establish the efficacy of different nanosystems acting as IFX nanocarriers. 

The use of nanocarriers is a growing field of research that may represent a powerful tool in the development of novel and more accurate therapies directed to RA. RA clinical management consists on the use of methotrexate (MTX) as a first line treatment or another disease-modifying antirheumatic drugs (DMARDs) as soon as diagnosis is confirmed. Combination therapy with biologic agents, mainly tumor necrosis factor inhibitors, are possible treatment options in RA [28,29]. IFX is an Ab targeting TNF-α and it is extensively used in a wide variety of pathologies, including RA [30]. A recent systematic review and meta-analysis conducted by Costa et al. [31] observed that the combination of IFX plus MTX results more effective than MTX alone or combined with other DMARDs, particularly during the initial period of treatment, as approximately 67% of patients finally produce anti-IFX antibodies after 18 months of therapy thereby limiting IFX response [32]. On the other hand, Siljehult et al. [33] demonstrated that the reduced response, rather than the presence of anti-IFX, is due to the lower levels of IFX in the patient. As higher dose of IFX could be related with increased risk of infections in RA patients, nanomedicine arises as a promising field in RA therapy. Through different nanosystems, drug delivery occurs specifically in the inflamed joint of patients with RA, acting either in immune or endothelial cells to maximize the success of the treatment received [34]. 

In this context, the use of dendrimers may provide a potential solution as targeted therapy in patients with RA [35]. Currently, seven dendrimers-based products are commercialized and another six derivates are being evaluated under clinical trials [36]. Thomas et al. [37] demonstrated the usefulness of a conjugated dendrimers with MTX and folate in both in vitro and in vivo models of RA. However, no dendrimers-antibody conjugates are approved or under clinical trial due to its important side effects, mainly cytotoxicity and immunoreactivity. Number of dendrimers, architecture, type and density of surface functional groups may be considered to deal with these important limitations [38,39]. Furthermore, dendrimers present multiple advantages to support their use. For instance, their uniform properties, biocompatibility, biodegradability, high drug loading capacity, water solubility, and the presence of multiple functional groups in its surface that may influence in the conjugation of multiple molecules at the same time. Marcinkowska et al. [40] also described that the branched structure of dendrimers prevent the premature release of antibody-conjugated drug, therefore increasing their performance. Likewise, dendrimers have been proven to increase the average life of antibodies in the bloodstream by improving the solubility and permeability of Abs, thus reducing the minimum therapeutic dose while maintaining proper levels of Abs, along with decreasing treatment-related side effects [41]. In this line, multiple studies have found better results in antibodies therapy when combined with dendrimers rather than free [42,43,44]. The function of dendrimers as nanocarriers of antibody permits a higher precision and efficacy in the targeted therapy, alone or in combination with added drugs [45,46,47]. Antibody-dendrimer conjugates have been shown in preclinical models to improve the targeting and release of chemotherapeutic drugs to the tumor while reducing secondary effects caused by drug accumulation in healthy tissues. This is the case of trastuzumab covalently linked to PAMAM dendrimer that accumulates in tumors overexpressing epidermal growth factor receptor 2 (HER-2) and only in small amounts in healthy tissues [48]. In this context, we must keep in mind that TNF-alpha, the target of IFX, increased in inflamed region in which it is attached to the membrane (mTNF-alpha) of inflammatory cells, so that the mechanism of action of IFX involves the induction of apoptosis of mTNF-alpha-overexpressing cells [49]. Unfortunately, there are no data in the literature on the distribution of antibody-dendrimer conjugates in experimental models of inflammation.

Our study is the first to combine a dendrimer with IFX, an TNF-α inhibitor antibody, which may be important to develop more precise, adequate therapies for patients with RA. Further research should be established to validate the role of this conjugate either in vitro or in vivo models. Moreover, we propose to bind additional treatments such as MTX or DMARDs before translation to reach the maximum benefits of this novel proposed targeted therapy.

## 4. Materials and Methods

Dendrimer G_3_Si(SC_2_H_4_NHFITC)(SC_2_H_4_CO_2_Na)_31_ was synthesized following the protocol described in the bibliography with the appropriate modifications described [50]. The procedure is described below. Solvents and the chemicals were purchased from commercial sources and used without prior treatment. Thiol-ene reactions were carried out using a UV lamp that radiates vertically (high efficiency with VL-115.L Viber Lourmat filter, 365 nm emission, 30 W, 1100 µW/cm^2^). NMR experiments were performed on Varian spectrometers, Mercury-300 or Unity-500 spectrometers or Bruker AV400, at ambient temperature. The chemical shifts (ppm) were measured relative to the residual signal of ^1^H of the deuterated solvents. Infrared spectra were measured in the IR-FT Perkin-Elmer Spectrum 2000, and ultraviolet spectra were taken from the UV-Vis spectrophotometer Perkin-Elmer Lambda 35.

### 4.1. Synthesis of G_3_Si(Allyl)_31_(SC_2_H_4_NH_2_HCl) (I)

To a solution of G_3_SiA_32_ (0.28 g, 0.07 mmol) [51] in a mixture of THF/MeOH (1:3) were added amineethane-2-thiol hydrochloride (0.0083 g, 0.07 mmol) and the photoinitiator DMPA (0.002 g, 0.007 mmol). The reaction was purged with a needle under argon for a minute and, subsequently, it was stirred for 30 min under ultraviolet light at room temperature. Without any further purification, the product was used to the next step. ^1^H-NMR (CDCl_3_): δ 8.41 (3H, s, N*H*_2_), 5.74 (31H, m, SiCH_2_C*H*CH_2_), 4.85 (62 H, m, SiCH_2_CHC*H*_2_), 3.22 (2H, t, *CH_2_*NH_2_), 2.92 (2 H, t, S*CH_2_*CH_2_NH_2_), 2.57 (2 H, t, SiCH_2_CH_2_*CH_2_*S), 1.56 (62 H, d, SiC*H*_2_CHCH_2_), 1.54 (2 H, m, SiCH_2_C*H*_2_CH_2_S), 1.33 (120 H, m, SiCH_2_C*H*_2_CH_2_Si), 0.57 (114 H, t, SiCH_2_), 0.05–−0.09 (84 H, s, SiMe).

### 4.2. Synthesis of G_3_Si(SC_2_H_4_NH_2_)(SCH_2_CO_2_CH_3_)_31_ (II)

Methyl tioglycolate (0.22 mL, 2.2 mmol) and DMPA (56 mg, 0.22 mmol) were added to the previous solution of I in THF/MeOH, and, after purging under argon for a minute, the reaction was stirred for 6 h under ultraviolet light at room temperature. A yellowish solution was obtained that was used without further purification for the next step. ^1^H-NMR (CDCl_3_): δ 3.69 (93 H, s, OC*H*_3_), 3.17 (62 H, s, SC*H*_2_CO), 2.59 (64 H, t, SiCH_2_CH_2_C*H*_2_S), 1.55 (62 H, m, SiCH_2_C*H*_2_CH_2_S), 1.23 (56 H, m, SiCH_2_C*H*_2_CH_2_Si), 0.53 (176 H, m, SiC*H*_2_), −0.08–−0.12 (84 H, s, SiC*H*_3_).

### 4.3. Synthesis of G_3_Si(SC_2_H_4_NH_2_)(SCH_2_CO_2_Na)_31_ (III)

To a solution of compound II was added sodium hydroxide (0.12 g, 3 mmol) solved in distilled water (60 mL aprox.) and stirred overnight at room temperature. Solvents were removed and the white powder were solved in distilled water and purified using a nanofiltration until pH = 7 (cellulose membranes of MWCO = 1000). Compound III was obtained as a white powder (447 mg, 92%). ^1^H-NMR (D_2_O): δ 3.09 (62 H, s, SCH_2_CO), 2.46 (64 H, m, SiCH_2_CH_2_CH_2_S), 1.50 (62 H, m, SiCH_2_CH_2_CH_2_S), 1.27 (56 H, m, SiCH_2_CH_2_CH_2_Si), 0.53 (176 H, m, SiCH_2_), −0.09 (84 H, s, SiCH_3_).

### 4.4. Synthesis of G_3_Si(SC_2_H_4_NHFITC)(SCH_2_CO_2_Na)_31_ (IV)

In a dark flask, compound III (12 mg, 0.0017 mmol) was solved in DMF and, subsequently, fluorescein isothiocyanate (FITC, 0.81 mg, 0.002 mmol) and triethylamine (0.4 mL, 0.0034 mmol) were added. The reaction was stirred overnight at room temperature and protected from the light. Compound IV was obtained as an orange powder after evaporating the solvents and washing with ethanol (10.9 mg, 83%). Kaiser test was performed to confirm there was not free amino group from free FITC (7732.49 g/mol). ^1^H-NMR (D_2_O): δ 6.7–6.9 (11 H, m, FITC), 3.22 (62 H, s, SCH_2_CO), 2.49 (64 H, m, SiCH_2_CH_2_CH_2_S), 1.51 (62 H, m, SiCH_2_CH_2_CH_2_S), 1.28 (56 H, m, SiCH_2_CH_2_CH_2_Si), 0.53 (176 H, m, SiCH_2_), −0.07 (84 H, s, SiCH_3_). IR (KBr): disappearance of isothiocyanate signal of free FITC υ (S=C=N–): 2180 cm^−1^. UV-Vis: 494 nm (FITC).

### 4.5. SDS-PAGE 

To separate the aggregates (dimers, trimers...), this experiment was performed using a polyacrylamide gel in denaturing and non-reducing conditions. To a solution of compound IV (0.2 µg/mL) in a final volume of 50 µL (PBS 1×, pH 7.3), it was added 10 µL of buffer solution 5×. Then, the solution was heated for five minutes at 95 °C. As weight marker, it was used ProSieve QuadColor Protein Markers (Lonza Rockland, Inc., Rockland, ME, USA). Electrophoresis experiment were carried out at 80 V for 3 h. Later, the gel was stained with blue Coomassie (0.4% blue Coomassie, 25% isopropanol, 10% acetic acid) for at least 1 h at room temperature and under stirring. After this time, the gel was washed five times with a washed solution (30% methanol, 10% acetic acid).

### 4.6. Formation of Dendriplex Infliximab-IV (IFX-IV)

The synthetic route to form the dendriplex is described in Figure 1. First of all, the carboxylate groups of the surface were activated using 2-(N-morpholino)ethanesulfonic (MES) 100 mM (pH 4.8) during 15 min, while stirring and protected from light. Afterwards, the coupling agents, N-hydroxysucinimide (C_4_H_5_NO_3_) (Thermo scientific, Waltham, MA, USA) and 1-ethyl-3-(3-dimethylaminopropyl) carbodiimide (EDCI) (Thermo scientific, Waltham, MA, USA), were added with a stoichiometry 1:16 (IV: C_4_H_5_NO_3_) and 1:8 (IV: EDCI), respectively. Instantly, the formation of an orange precipitate was observed. Again, the reaction was stirred and protected from the light for 1 h. After this time, the reaction was centrifuged for 10 min at 19,000× *g*. The supernatant was removed and the orange pellet was solved in HEPES at 100 mM, in which infliximab (Remicade, Centocor BV, Leiden, The Netherlands) was incorporated at different ratios (IV: IFX; 1:1, 2:1, 5:1 and 10:1). The reaction was stirred for 24 h at 4 °C and protected from the light. Partial purification was carried out by dialysis, using a Slide-A-Lyzer Dialysis cassette 10,000 MWCO (Thermo Scientific, Waltham, MA, USA) for 24 h at 4 °C in the dark and changing the medium (HEPES 100 mM) three times. Then, the solution was subject to Sephadex G25 column chromatography (Thermo Fisher Waltham, MA, USA) at a flow rate of 2 mL/min in PBS (1×, pH 7.3) at 4 °C in the dark. Eighty fractions of 500 μL were collected, in order to purify the dendriplex. The fluorescence of the fractions was determined at a wavelength of 494 nm using a Victor2 D fluorometer system (Perkin Elmer, Waltham, MA, USA).

The dendrimer:IFX ratio used was determined carrying out the same experiment at dendrimer:AB ratios 1:1, 2:1, 5:1, and 10:1; only the 5:1 ratio showed the binding of at least one dendrimer per IFX.

### 4.7. Characterization of the Dendriplex by SE-HPLC, SDS-PAGE and Inmunoblot

The purity of the fractions previously obtained was checked by SE-HPLC chromatography. Briefly, 50 µL of the sample was injected on a YARRA3000 (Phenomenex) column installed on the Agilent Technologies 1200 series HPLC (Agilent Technologies). Chromatography was performed in PBS (1×, pH 7.3) with a flow of 0.350 mL/min for 18 min. Fluorescence (494 nm) and ultraviolet (280 nm) detectors were used. 

For SDS-PAGE characterization and subsequent inmunoblotting, a mixed polyacrylamide gel was used under non-reducing and denaturing conditions. Two solutions of 0.2 and 0.4 µg/mL of the dendriplex IFX-IV were prepared, and a 5× sample buffer was added. Pure infliximab (0.2 µg/µL) was used as a positive control and compound IV (0.2 μg/μL) as a negative control, both dissolved in sample buffer 5×. All the samples were heated at 95 °C for 5 minutes. ProSieve QuadColor Protein Markers (Lonza Rockland, Inc., Rockland, ME, USA) was used for the determination of the molecular weight of IFX-IV. 

Once electrophoresis was concluded, the transfer was made to a PVDF membrane. For this, the transfer kit was prepared with Wattman paper and left overnight at 25 V. The PVDF membrane was stained with Ponceau Red and then washed 3 times for 5 min each with TTBS (0.05% tween 20). Subsequently, the membrane was incubated under stirring with Anti-Mouse IgG (1:6000) in TTBS (1.05% tween 20) for 1 h. After that time, it was washed with TTBS (0.05% tween 20) 6 times, changing the medium every 5 min and with stirring. Detection was carried out by the incubation of the membrane with chemiluminescent reagent (Amersham ECL Western Blotting Detection Reagents, Marlborough, MA, USA) and subsequent exposure to an autoradiographic film (Hyperfilm ECL, Amersham, The Netherlands).

### 4.8. Functional Characterization of Dendriplex IFX-IV by ELISA

The IFX-IV concentration obtained was assessed using the Duo Infliximab LISA-TRACKER Kit (Theradiag, Croissy Beaubourg, France). For this, different dilutions of IFX-IV were incubated for 1 h at room temperature in 96-well plates that had TNF-α on their surface. After that time, they were washed three times with wash buffer and then incubated with a biotinylated antibody anti-Infliximab for 1 h at room temperature. Then, they were washed again three times with washing buffer. Later, they were incubated with strepravidin HRP conjugate for 30 min at room temperature. They were washed three times with washing buffer. Then, they were incubated with tetramethylbenzidine (TMB) for 15 min in dark conditions. After this, the reaction was stopped by adding sulfuric acid (0.25 M), and the absorbance at 450 nm was measured before 30 min. The absorbance values were compared with the standard curve of IFX (µg/mL). As a negative control, IV was used. In addition, the number of dendrimers coupled to IFX was determined using the protocol proposed by Thermo Scientific (calculate dye: protein (F/P) molar ratios) [52].

## 5. Conclusions

Nanotechnology and particularly dendrimers are powerful approaches to maximize the efficacy of current treatments in RA, including biological and Ab therapy. For the first time, we have developed a stable conjugated carbosilane dendrimer with IFX. Although it still lacks scientific evidence to support its use, the multiple benefits of dendrimers and other nanosystems and nanocarriers makes future research worth performing. This study will support the basis of further research related to dendrimers-Ab conjugates in RA, with the possibility of combining additional treatments for future in vitro and in vivo models.
